# Pulmonary function before and after the Nuss procedure in adolescents with pectus excavatum: correlation with morphological subtypes

**DOI:** 10.1186/s13019-015-0236-7

**Published:** 2015-03-22

**Authors:** Jin Yong Jeong, Joong Hyun Ahn, Sang Yong Kim, Yoon Hong Chun, Kyungdo Han, Sung Bo Sim, Keon Hyon Jo

**Affiliations:** 1 Department of Thoracic and Cardiovascular Surgery, Incheon St. Mary’s Hospital, College of Medicine, The Catholic University of Korea, Seoul, Republic of Korea; 2 Department of Internal Medicine, Incheon St. Mary’s Hospital, College of Medicine, The Catholic University of Korea, Seoul, Republic of Korea; 3 Department of Pediatrics, Incheon St. Mary’s Hospital, College of Medicine, The Catholic University of Korea, 222 Banpo-daero, Seocho-gu, Seoul, Republic of Korea; 4 Department of Biostatistics, The Catholic University of Korea, Seoul, Republic of Korea; 5 Department of Thoracic and Cardiovascular Surgery, Yeouido St. Mary’s Hospital, College of Medicine, The Catholic University of Korea, Seoul, Republic of Korea; 6 Department of Thoracic and Cardiovascular Surgery, Seoul St. Mary’s Hospital, College of Medicine, The Catholic University of Korea, Seoul, Republic of Korea

**Keywords:** Pectus excavatum, Pulmonary function test, Adolescents

## Abstract

**Background:**

Differences in post-Nuss procedure pulmonary function based on the pectus excavatum subtype have not been investigated in adolescents. We evaluated differences in pulmonary function before and after the Nuss procedure according to preoperative morphology.

**Methods:**

We performed a retrospective review of eighteen male patients who had undergone the Nuss procedures. There were nine patients each with symmetric and asymmetric morphology. Patients were younger than 18 years and had no history of respiratory diseases. Pulmonary function was assessed 2 weeks before and 4–6 months after the surgery. Preoperative and postoperative pulmonary function data were compared between the symmetric and asymmetric types. The paired *t*-test was used to compare the differences within each group and an analysis of covariance (ANCOVA) was used to access intergroup differences.

**Results:**

There were no significant demographic differences between patients with symmetric and asymmetric subtypes. Patients with the asymmetric type had a lower preoperative total lung capacity (TLC) (*p* = 0.018), vital capacity (VC) (*p* = 0.0308), and inspiratory capacity (IC) (*p* = 0.0373). In both types, the forced vital capacity (FVC), forced expiratory volume in one second (FEV1), and VC were all significantly decreased postoperatively compared to baseline (all, *p* < 0.01). The asymmetric type showed further reductions in peak expiratory flow (PEF) (*p* = 0.0391) and IC (*p* = 0.0084) postoperatively. The residual volume (RV) (*p* = 0.0092) and RV/TLC ratio (*p* = 0.0025) increased significantly in the asymmetric type, but only the postoperative PEF values differed significantly between the two types (*p* = 0.0151).

**Conclusions:**

The asymmetric type had poorer preoperative lung volumes and poorer postoperative pulmonary function, with significantly lower PEF compared to the symmetric type cases. Preoperative and postoperative lung function needs more careful evaluation until pectus bar removal in the asymmetric type of pectus excavatum.

## Background

Pectus excavatum, the most common congenital chest wall deformity of childhood, can cause cosmetic problems, as well as exercise intolerance, fatigue, dyspnea, and chest pain [1]. In 1998, Nuss introduced a new minimally invasive procedure that has since become the standard technique for pectus excavatum repair [2-5].

Despite multiple investigations [1,2,6,7], there is has been no consensus regarding the effect of the Nuss procedure on pulmonary function. One study evaluating pulmonary function in pediatric patients showed a significant decline in the forced vital capacity (FVC) and vital capacity (VC), but not in the forced expiratory volume in 1 second (FEV_1_) or total lung volume (TLV) shortly after the Nuss procedure with the bar still in place [6]. Another study of patients of similar age and with the pectus bar in place reported no significant change in the FVC, FEV1, forced expiratory flow between 25% and 75% of expired vital capacity (FEF_25–75_), and total lung capacity (TLC) after the first stage of the Nuss procedure [7].

Several studies have reported on postoperative pulmonary function, but show conflicting outcomes. The preoperative data in some studies show that morphologic variants and upper thoracic shape in children with pectus excavatum can affect pulmonary function [1,8]. However, we could not find any study that evaluated the effect of morphological subtypes of pectus excavatum on preoperative and postoperative pulmonary function. We hypothesized that the subtypes of pectus excavatum could be the cause of differences in pulmonary function measurements before and after the Nuss procedure.

## Methods

From February 2012 to February 2013 in our hospital, 18 male adolescents underwent pectus excavatum repair by the Nuss procedure. All patients were younger than 18 years and had no unrelated respiratory disease. Preoperative evaluation included a computed tomography (CT) scan of the chest and pulmonary function tests. This study was based on a retrospective chart review of these patients and was approved by the Institutional Review Board of Incheon St. Mary’s Hospital College of Medicine at the Catholic University of Korea (Seoul, Republic of Korea).

### Surgical technique

The procedure was performed in a similar manner as described in a previous article [9]. Under general anesthesia, the patient was placed in the supine position with both arms abducted. A crane was routinely applied to the depressed sternum at the xiphoid to elevate the sternum. Tiny skin incisions were opened on either side of the chest, and a subcutaneous tunnel was formed. Hinge points were created. After dissecting the retrosternal space using a needlescope (2-mm Mini-fiber telescope®; Richard Wolf, Ltd., Knittlingen, Germany) and endoscissors (Richard Wolf, Ltd.), a 20 Fr chest tube was inserted through one incision and passed across the midline and out the other incision with the pectus clamp [10]. The pectus bar was inserted with the guidance of the chest tube and then rotated. The bar was fixed to the reciprocal ribs on both sides with the fixator and anterior chest wall with No. 5 Ethibond (Ethibond Excel, Ethicon Inc., Somerville, NJ, USA) sutures using a needlescope-assisted 3 three-point fixation method [11]. This surgical technique has been described in detail in previous publications [9-11].

### Morphological classification of pectus excavatum

Based on the axial plane of the CT scans, we divided the chest wall deformities into “symmetric type” or “asymmetric type”. In the symmetric type, the center of the sternum and the center of the depression are congruent. In the asymmetric type, the center of the depression is not located in the center of the sternum but is off to either side [9].

### Pulmonary function testing

Pulmonary function was assessed 2 weeks before surgery and 4–6 months after surgery, with the bar still in place. All spirometry and plethysmography findings were analyzed by a single pulmonologist. The following dynamic flow rates and static lung volumes were examined: FVC, FEV_1_, FEF_25–75_, peak expiratory flow (PEF), maximal voluntary ventilation (MVV), TLC, VC, inspiratory capacity (IC), and residual volume (RV). FVC and FEV_1_ values of less than 80% predicted and an FEV_1_/FVC ratio greater than 80% were considered a restrictive pattern; and an FEV1/FVC ratio of less than 67% were considered an obstructive pattern [8]. To exclude the effect of growth on lung volumes, all pulmonary function values were expressed as a percentage of the predicted value for sex, age, and height (mean percentage of normal values ± standard deviation [SD]). Raw dynamic flow rates were normalized using Knudson’s equations [12].

### Haller index

The CT scans were used to determine the Haller index. The Haller index was calculated as the inner transverse thoracic diameter divided by the anteroposterior distance between the anterior thoracic wall and the spine at the narrowest point [13].

### Statistical analysis

All data are expressed as the mean ± the SD or by the number of patients affected. The categorical variables were analyzed using Fisher’s exact test. Continuous variables were analyzed using the independent *t*-test. Differences between preoperative and postoperative values within each group were performed using the paired *t*-test. Analysis of covariance (ANCOVA) with age and baseline pulmonary function test (PFT) adjustment was used to compare the differences between the two groups in postoperative PFT changes. All statistical analyses were performed using SAS for Windows, release 9.2 software (SAS Institute, Cary, NC, USA). Results were considered statistically significant at *p* < 0.05.

## Results

The demographic features of children who underwent pectus excavatum repair by the Nuss procedure are summarized in Table 1. Of the 18 patients, half had symmetric pectus excavatum and the other half had asymmetric pectus excavatum. There were no significant differences between the mean age, weight, height and body-mass index (BMI) of patients with the two types of pectus excavatum. In the preoperative PFTs, only one patient with the asymmetric type had the restrictive pattern, and no patient in either group had the obstructive pattern. However, after the procedure, one patient with the symmetric type and four patients with the asymmetric type had the restrictive pattern. The Haller indices showed no significant differences in the changes between baseline and postoperative values between the two groups (Table 2).

**Table 1:** **Patient characteristics**

	Symmetric type (*n* = 9)	Asymmetric type (*n* = 9)	*p*
Male, *n* (%)	9 (100)	9 (100)	1
Age (y)	14.3 ± 1.6	14.2 ± 2.4	0.910
Weight (kg)	54.4 ± 11	54 ± 8.9	0.926
Height (m)	1.7 ± 0.1	1.7 ± 0.1	0.799
BMI (kg/m^2^)	18.6 ± 1.9	18.9 ± 2.2	0.781

BMI = body-mass index.

**Table 2:** **Changes in the pulmonary function and Haller index after the bar insertion**

	Symmetric type (*n* = 9)	Asymmetric type (*n* = 9)	*p*
Preoperative PF			
Restrictive, *n* (%)^a^	0 (0)	1 (5.6)	1
Obstructive, *n* (%)^b^	0 (0)	0 (0)	1
Postoperative PF			
Restrictive, *n* (%)	1 (5.6)	4 (22.2)	0.294
Obstructive, *n* (%)	0 (0)	0 (0)	1
Preoperative HI^c^	3.8 ± 0.8	3.8 ± 1	0.971
Postoperative HI	2.7 ± 0.4	2.8 ± 0.2	0.387

HI = Haller index; PF = pulmonary function.

^a^The restrictive pattern is forced volume capacity (FVC) and forced expiratory volume in 1 second (FEV_1_) values of less than 80% predicted and an FEV_1_/FVC ratio greater than 80%.

^b^The obstructive pattern is a FEV1/FVC ratio of less than 67%.

^c^The Haller index is calculated as the inner transverse thoracic diameter divided by the anteroposterior distance between the anterior thoracic wall and the spine at the narrowest point.

In the preoperative lung volume data, the asymmetric type had a lower TLC (*p* = 0.018), VC (*p* = 0.0308), and IC (*p* = 0.0373) (Table 3). Table 4 shows the PFT changes after the pectus bar insertion. The FVC and FEV1 values significantly decreased in both types (for all, *p* < 0.01). The FEV1/FVC ratio increased significantly in both types (for all, *p* < 0.01). After the pectus bar insertion, the PEF values significantly decreased only in the asymmetric type (*p* = 0.0391); in contrast, the MVV significantly increased only in the symmetric type (*p* = 0.0397). The TLC in both types showed no difference between the preoperative and postoperative values. However, the VC values in both types decreased significantly after the pectus bar insertion (for all, *p* < 0.01). After the procedure, the asymmetric type had a significant decrease in the IC value (*p* = 0.0084). In addition, the RV value (*p* = 0.0092) and RV/TLC ratio (*p* = 0.0025) increased significantly only in the asymmetric type. When comparing both types of pectus excavatum after the procedure, the percent changes in the PFTs were similar, except for the PEF values (Table 4). When ages and baseline PFT values were adjusted, the asymmetric type showed a significant decrease in the PEF, compared to the symmetric type (*p* = 0.0151).

**Table 3:** **Preoperative spirometry and lung volume data**

	Symmetric type	Asymmetric type	*p*
FVC	106.3 ± 12.6	97.4 ± 13.5	0.1691
FEV_1_	104 ± 10.4	96.4 ± 13.9	0.2101
FEV_1_/FVC	105.1 ± 7.4	105.9 ± 4.3	0.7882
FEF_25–75_	114.4 ± 19.3	103.8 ± 18.8	0.2521
PEF	111 ± 14.4	108.1 ± 14	0.6714
MVV	86 ± 15.1	83.8 ± 13.2	0.7431
TLC	115.8 ± 12.2	96.7 ± 14.2	0.018*
VC	112.7 ± 7.1	96.7 ± 15	0.0308*
IC	74.3 ± 12	58.7 ± 13.3	0.0373*
RV	134.2 ± 39.6	101.2 ± 24.9	0.0678
RV/TLC	114.2 ± 23.3	105.2 ± 19.2	0.431

FEF_25–75_ = forced inspiratory flow from 25% exhalation to 75% exhalation; FEV1 = forced expiratory volume in 1 second; FVC = forced vital capacity; IC = inspiratory capacity; MVV = maximum voluntary ventilation; PEF = peak expiratory flow; RV = residual volume; TLC = total lung capacity; VC = vital capacity.

*Values of *p* < 0.05 are statistically significant.

**Table 4:** **Spirometry and lung volume changes after the Nuss procedure**

	Symmetric type	*p* ^a^	Asymmetric type	*p* ^a^	*p* ^b^
FVC	−20.3 ± 5.2	<0.0001*	−18.8 ± 9.9	0.0005*	0.8671
FEV_1_	−15.3 ± 6.6	0.0001*	−15.6 ± 10.4	0.002*	0.8012
FEV_1_/FVC	5.4 ± 4.7	0.0081*	4.7 ± 4	0.0078*	0.8656
FEF_25–75_	9.4 ± 22.6	0.2445	10.2 ± 10.3	0.0178*	0.9135
PEF	3.3 ± 9.6	0.3282	−8.1 ± 9.9	0.0391*	0.0151*
MVV	8.1 ± 9.9	0.0397*	7.4 ± 20.8	0.3134	0.9249
TLC	−7.2 ± 12	0.2525	−6.6 ± 9.1	0.0626	0.9083
VC	−20.2 ± 7.3	0.0035*	−18 ± 11.7	0.0017*	0.6916
IC	−18.8 ± 20.2	0.1064	−16.4 ± 14.2	0.0084*	0.2269
RV	41.8 ± 64.5	0.2211	37.4 ± 32.9	0.0092*	0.9985
RV/TLC	41.6 ± 41.8	0.0903	48.6 ± 33.6	0.0025*	0.8341

FEF_25–75_ = forced inspiratory flow from 25% exhalation to 75% exhalation; FEV1 = forced expiratory volume in 1 second; FVC = forced vital capacity; IC = inspiratory capacity; MVV = maximum voluntary ventilation; PEF = peak expiratory flow; RV = residual volume; TLC = total lung capacity; VC = vital capacity.

^a^The *p* values of the difference between the postoperative parameters and the preoperative parameters.

^b^The *p* values of the difference between the symmetric type and the asymmetric type.

*Values of *p* < 0.05 are statistically significant.

## Discussion

Postoperative pulmonary function shows a long-term improvement after surgical correction of pectus excavatum [14]. However, the recently developed Nuss procedure, which relies on internal bracing for 2 to 4 years to reform the chest wall, has shown conflicting results with regard to pulmonary function in growing adolescents. Our results suggest that morphological differences in pectus excavatum can be responsible for conflicting pulmonary function changes in postoperative patients with the bar still in place.

The most common effect of chest wall deformities on the PFTs is the gradual development of a restrictive lung pattern that is characterized by decreased TLC [15]. In the preoperative data, patients with the asymmetric type showed lower TLC, VC, and IC, compared to patients with the symmetric type, although the Haller indices of both types were not different. The PFTs of one patient with the asymmetric type showed a frankly restrictive pattern. As a result, the asymmetric type had unfavorable PFTs before the Nuss procedure, compared to the symmetric type. In addition, our results showed that the Haller index does not reflect the effect of the morphological varieties of pectus excavatum, although it is a clinical and practically useful measure.

After the Nuss procedure, our results showed significantly decreased FVC, FEV_1_, and VC values in both groups. A previous study examined the early postoperative effects of Nuss repair without considering morphological differences and reported similar results in adolescents [6]. Sigalet et al. studied the pulmonary function of patients before and 3 months after the Nuss operation. They demonstrated reduced dynamic pulmonary function, FVC, FEV1, and VC after the surgery. Our study, which took into account morphological differences, demonstrated a greater reduction of PEF and IC values in the asymmetric type. Further, the number of patients who had restrictive-type PFTs in the asymmetric type increased from one patient to four patients after the bar insertion. This discrepancy between symmetric and asymmetric types may explain the conflicting results of postoperative lung function in the literature over the years.

When we compared the changes in pulmonary function data between the two types, only the PEF values showed a significant difference after the Nuss procedure. Decreased PEF in the asymmetric type can be associated with the increased RV and RV/TLC ratio after Nuss bar insertion. An increased RV can be induced by chest wall defects that interfere with the lungs’ return to their neutral position and do not allow expiratory muscles to produce a full exhalation because of the mechanical disadvantage [15]. The metallic bar inserted in the thorax of pectus excavatum patients can lessen the movement of the bony thorax and the expiratory muscles. Some patients with the asymmetric type needed the insertion of more than one pectus bar in our study. It is also possible that correcting the asymmetry of the two hemithoraces that contain one hyperinflated lung and one hypoinflated lung can reduce the expiratory flow rate. The asymmetric type showed decreased IC and VC after the bar insertion. This may be because the smaller hemithorax does not fully recover to compensate for the reduction in the larger hemithorax.

A meta-analysis that assessed pulmonary function recovery reported that the Nuss procedure was correlated with greater improvements in pulmonary function after bar removal, depending on the time after the operation [14]. In a long-term follow-up study, Sigalet et al. reported a significant increase in FEV_1_ and TLC after bar removal; however, they concluded that the pectus bar did not improve pulmonary dynamics shortly after the operation [6,16]. These favorable outcomes suggest that the lung function parameters of our patients may improve after bar removal. However, the time needed for the steel strut to properly reform the chest is not short in adolescents who are undergoing periods of growth. The thoracic volume increases to approximately 50% of the adult size by 10 years of age and the remaining 50% develops during early adolescence [17]. There is a need for better observation of pulmonary function and timely intervention in patients who undergo rapid growth in the thoracic cavity and in the intrathoracic organs.

The present study has some limitations. We could not compare pulmonary function changes with baseline measurements more than once after the Nuss procedure. Some studies did not consider morphological differences, although some studies showed significant improvement in follow-up PFTs performed 8 months or 12 months postoperatively [18,19]. To determine the influence of morphological differences on pulmonary function recovery, repeated studies of lung function tests at various times are needed. Another limitation of our study is that we did not measure exercise tolerance after the Nuss procedure. The actual functional outcomes of the procedure can differ from the decreased pulmonary dynamics because cardiovascular function also changes postoperatively. Further studies that include cardiopulmonary function during exercise are also needed to clarify our results.

## Conclusions

We demonstrated that morphological differences in pectus excavatum may affect pulmonary function before and after the Nuss procedure. In particular, patients with the asymmetric type can have a disadvantage in preoperative lung volume and postoperative pulmonary function, compared to patients with the symmetric type. Our results suggest that a more careful evaluation of preoperative and postoperative pulmonary function may be useful in patients with pectus excavatum, especially in the asymmetric type.

### Consent

Written informed consent was obtained from the patient’s parent for the publication of this report and any accompanying images.

## References

[1] Redding GJ, Kuo W, Swanson JO, Phillips GS, Emerson J, Yung D (2013). Upper thoracic shape in children with pectus excavatum: impact on lung function. Pediatr Pulmonol.

[2] Aronson DC, Bosgraaf RP, Merz EM, van Steenwijk RP, van Aalderen WM, van Baren R (2007). Lung function after the minimal invasive pectus excavatum repair (Nuss procedure). World J Surg.

[3] Nuss D, Kelly RE, Croitoru DP, Katz ME (1998). A 10-year review of a minimally invasive technique for the correction of pectus excavatum. J Pediatr Surg.

[4] Uemura S, Nakagawa Y, Yoshida A, Choda Y (2003). Experience in 100 cases with the Nuss procedure using a technique for stabilization of the pectus bar. Pediatr Surg Int.

[5] Pilegaard HK, Licht PB (2008). Early results following the Nuss operation for pectus excavatum—a single-institution experience of 383 patients. Interact Cardiovasc Thorac Surg.

[6] Sigalet DL, Montgomery M, Harder J (2003). Cardiopulmonary effects of closed repair of pectus excavatum. J Pediatr Surg.

[7] Borowitz D, Zallen G, Sharp J, Burke M, Gross K, Glick PL (2003). Pulmonary function and response to exercise following Nuss repair in patients with pectus excavatum. J Pediatr Surg.

[8] Lawson ML, Mellins RB, Paulson JF, Shamberger RC, Oldham K, Azizkhan RG (2011). Increasing severity of pectus excavatum is associated with reduced pulmonary function. J Pediatr.

[9] Park HJ, Jeong JY, Jo WM, Shin JS, Lee IS, Kim KT (2010). Minimally invasive repair of pectus excavatum: a novel morphology-tailored, patient-specific approach. J Thorac Cardiovasc Surg.

[10] Jeong JY, Lee J (2014). Use of needlescope and crane technique to avoid cardiac injury in Nuss procedure. Ann Thorac Surg.

[11] Jeong JY, Lee J (2014). Needlescope-assisted 3-point fixation of the pectus bar in the Nuss procedure. J Thorac Cardiovasc Surg.

[12] Knudson RJ, Lebowitz MD, Holberg CJ, Burrows B (1983). Changes in the normal maximal expiratory flow-volume curve with growth and aging. Am Rev Respir Dis.

[13] Lawson ML, Barnes-Eley M, Burke BL, Mitchell K, Katz ME, Dory CL (2006). Reliability of a standardized protocol to calculate cross-sectional chest area and severity indices to evaluate pectus excavatum. J Pediatr Surg.

[14] Chen Z, Amos EB, Luo H, Su C, Zhong B, Zou J (2012). Comparative pulmonary functional recovery after Nuss and Ravitch procedures for pectus excavatum repair: a meta-analysis. J Cardiothorac Surg.

[15] Koumbourlis AC (2014). Chest wall abnormalities and their clinical significance in childhood. Paediatr Respir Rev.

[16] Sigalet DL, Montgomery M, Harder J, Wong V, Kravarusic D, Alassiri A (2007). Long term cardiopulmonary effects of closed repair of pectus excavatum. Pediatr Surg Int.

[17] Gomez JA, Lee JK, Kim PD, Roye DP, Vitale MG (2011). “Growth friendly” spine surgery: management options for the young child with scoliosis. J Am Acad Orthop Surg.

[18] Quigley PM, Haller JA, Jelus KL, Loughlin GM, Marcus CL (1996). Cardiorespiratory function before and after corrective surgery in pectus excavatum. J Pediatr.

[19] Tang M, Nielsen HH, Lesbo M, Frøkiær J, Maagaard M, Pilegaard HK (2012). Improved cardiopulmonary exercise function after modified Nuss operation for pectus excavatum. Eur J Cardiothorac Surg.

